# Sailing the Seven Seas: A Multinational Comparison of ChatGPT’s Performance on Medical Licensing Examinations

**DOI:** 10.1007/s10439-023-03338-3

**Published:** 2023-08-08

**Authors:** Michael Alfertshofer, Cosima C. Hoch, Paul F. Funk, Katharina Hollmann, Barbara Wollenberg, Samuel Knoedler, Leonard Knoedler

**Affiliations:** 1 Division of Hand, Plastic and Aesthetic Surgery, Ludwig-Maximilians University Munich, Ziemssenstrasse 5, 80336 Munich, Germany; 2 Department of Otolaryngology, Head and Neck Surgery, School of Medicine, Technical University of Munich (TUM), Ismaningerstrasse 22, 81675 Munich, Germany; 3 Department of Otolaryngology, Head and Neck Surgery, University Hospital Jena, Friedrich Schiller University Jena, Am Klinikum 1, 07747 Jena, Germany; 4 Department of Pathology, Massachusetts General Hospital, Harvard Medical School, 55 Fruit St, Boston, MA 02114 USA; 5 Department of Plastic, Hand and Reconstructive Surgery, University Hospital Regensburg, Franz-Josef-Strauss-Allee 11, 93053 Regensburg, Germany

**Keywords:** ChatGPT, OpenAI, Artificial intelligence, Medical education, Clinical decision-making, Medical licensing exams

## Abstract

**Purpose:**

The use of AI-powered technology, particularly OpenAI’s ChatGPT, holds significant potential to reshape healthcare and medical education. Despite existing studies on the performance of ChatGPT in medical licensing examinations across different nations, a comprehensive, multinational analysis using rigorous methodology is currently lacking. Our study sought to address this gap by evaluating the performance of ChatGPT on six different national medical licensing exams and investigating the relationship between test question length and ChatGPT’s accuracy.

**Methods:**

We manually inputted a total of 1,800 test questions (300 each from US, Italian, French, Spanish, UK, and Indian medical licensing examination) into ChatGPT, and recorded the accuracy of its responses.

**Results:**

We found significant variance in ChatGPT’s test accuracy across different countries, with the highest accuracy seen in the Italian examination (73% correct answers) and the lowest in the French examination (22% correct answers). Interestingly, question length correlated with ChatGPT’s performance in the Italian and French state examinations only. In addition, the study revealed that questions requiring multiple correct answers, as seen in the French examination, posed a greater challenge to ChatGPT.

**Conclusion:**

Our findings underscore the need for future research to further delineate ChatGPT’s strengths and limitations in medical test-taking across additional countries and to develop guidelines to prevent AI-assisted cheating in medical examinations.

**Dear Editor,**

ChatGPT and the phalanx of chatbots have aroused public hype and scientific interest. The next-generation of artificial intelligence (AI)-powered technology carries the potential to revolutionize healthcare and medical education [1–3].

There is a mounting body of evidence underscoring ChatGPT’s promising test-taking performance. Our group showed that ChatGPT answered 57.3% of otolaryngology/head and neck surgery board certification preparation questions correctly in a large dataset of *n* = 2,576 questions [4]. Recently, numerous groups analyzed ChatGPT’s performance on national medical licensing examinations including the Japanese, Chinese, or German medical state examination [5–7].

While such findings may herald a dogmatic shift in generating and evaluating medical test questions, their scientific significance remains to be elucidated. Our current understanding of ChatGPT’s test taking capabilities is mainly derived from mononational studies assessing a heterogenous and/or unrepresentative set of test questions. To this date, there is a paucity of studies providing a multinational overview and comparison of ChatGPT’s medical test-taking performance based on a rigorous methodology.

Herein, we aimed to determine ChatGPT’s test performance on six different national medical licensing examinations and compare the overall test accuracy based on test language and location. This line of research may provide another puzzle piece in understanding and leveraging the use of AI-based large language models such as ChatGPT.

## Methods

From June 22, 2023, to June 29, 2023, we accessed the question bank AMBOSS© (New York, NY, USA) and randomly extracted 300 United States Medical Licensing Examination (USMLE®) Step 2CK practice questions. Further, we randomly selected 300 freely accessible test questions each from Esame di Stato (Italian medical licensing examination), Examen National Classant (French medical licensing examination), Examen Medico Interno Residente (Spanish medical lincensing examination), Professional and Linguistic Assessments Board Exam (UK medical licensing examination), and the Foreign Medical Graduates Examination (Indian medical licensing examination). Prior to the initiation of the study, official permission for the use of the AMBOSS© USMLE® Step 2CK practice question bank for research purposes was granted by AMBOSS© (Amboss GmbH, Berlin, Germany). To assess ChatGPT’s capabilities in answering multiple-choice questions correctly, the evaluation of the Examen National Classant (French medical licensing examination)—which is designed in a multiple-choice test question design—was simplified to a dichotomous (i.e., 0 or 1) scoring system. This decision was made due to limited publicly available information on specific evaluation criteria, particularly regarding partial credit for partially correct answers. By adopting this straightforward approach, the authors aimed to focus on ChatGPT’s ability to identify all correct answers and distinguish them from incorrect ones in a multiple-choice format. This allowed for maximum comparability of ChatGPT’s performance on the French medical state examination with the other national licensation examinations included in this study, which are all designed in a single-choice test question design. All sample test questions were screened independently by four investigators (M.A.; S.K.; C.C.H.; L.K.), and questions including clinical images and photographs were removed. One investigator (L.K.) then manually entered the test questions into ChatGPT (OpenAI, San Francisco, CA, USA).

The test questions were manually inputted into ChatGPT, warranting an exact replication of the original question text and answer choices. To maintain the integrity of ChatGPT performance, the authors consciously refrained from introducing any supplementary prompts, thereby mitigating the potential for systematic errors. For each question, a fresh chat session was initiated in ChatGPT to minimize the influence of memory retention bias.

Responses from ChatGPT were recorded and entered into the corresponding test question. Subsequently, data regarding the accuracy of the responses and test question length (in characters) was collected in a separate dedicated spreadsheet.

Statistical analysis was conducted with IBM® SPSS Statistics Version 25 (Armonk, NY, USA), and a two-tailed *p* value of ≤ 0.05 was deemed to indicate statistical significance.

## Results

### General Test Question Characteristics and Performance Statistics

**Fig. 1:**
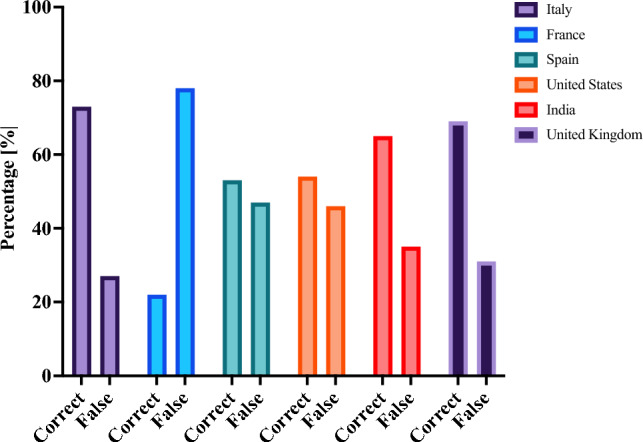
ChatGPT’s performance for different national medical licensing examinations.

For each medical licensing examination, a total of *n* = 300 test questions was manually entered into ChatGPT. ChatGPT’s test accuracy varied significantly across different countries (*p* < 0.001). ChatGPT’s performance was most accurate for the Italian medical licensing examination (73% correct answers, *n* = 220) but performed poorest when answering the French medical licensing examination (22%, *n* = 67). (Fig. 1) Mean test question length was 194 ± 64 characters for the Indian, 307 ± 113 for the Italian, 381 ± 169 for the French, 444 ± 142 for the UK, 532 ± 220 for Spain, and 726 ± 179 characters for the US medical licensing examination. Test question length was significantly different across the countries included (*p* < 0.001).

### Test Question Length and ChatGPT Performance

Test question length significantly correlated with ChatGPT’s performance in the Italian (*r*_s_ =  − 0.178; *p* = 0.002) and French medical licensing examination (*r*_s_ =  − 0.115; *p* = 0.046). In the Italian medical licensing examination, mean test question length was 336 ± 117 for the incorrect and 296 ± 109 for the correct answers (*p* = 0.006). In the French medical licensing examination, mean test question length was 393 ± 169 for incorrectly and 341 ± 162 for correctly answered test questions (*p* = 0.026).

## Discussion

This study is the to-date first effort to directly compare ChatGPT’s test-taking performance on a global level. Our analysis including 1,800 medical licensing examination questions from six different countries revealed great international variability regarding accuracy levels. While ChatGPT scored 73% when taking the Italian medical licensing examination, we found significantly lower accuracy levels for the French medical licensing examination (22%). Interestingly, the French examination is characterized by a unique test design as it requires examinees to select multiple correct answers for each test question (i.e., multiple-choice test question design). Our group has proposed different evidence-based strategies (e.g., level of difficulty, buzz words, test question style) to prevent AI-cheating in career-deciding exams such as the USMLE® Step 2CK. (unpublished data) ChatGPT’s poor performance in the French medical licensing examination points toward another lever (i.e., multiple correct answers instead of a single correct answer for each test question) to counteract AI-cheating, warranting further in-depth research.

In a previous study, we identified test question length (measured in characters) as a reliable surrogate parameter to predict ChatGPT’s performance in USMLE® Steps. (unpublished data) Surprisingly, test question length correlated with ChatGPT’s test performance only in the Italian and French medical licensing examinations, yielding significantly higher character counts for incorrect answers. This finding calls for specific evaluations of ChatGPT’s strengths and weak points in test-taking. While test-question length apparently represents a promising avenue for preventing AI-cheating in some state examinations, this parameter seems to be of less significance for ChatGPT’s accuracy in other examinations.

## Conclusion

This study provided a multinational and novel insight into ChatGPT’s test-taking abilities. We demonstrated that ChatGPT’s performance was poorest when answering questions that required multiple correct answers, as seen in the French medical licensing examination. Further, mean test question length did not represent a universal parameter to predict and potentially influence ChatGPT’s performance. Ultimately, future in-depth research is warranted to expand such investigations onto additional countries, ultimately establishing all-embracing and country-specific guidelines to thwart AI-cheating in medical licensing examinations.
